# Scalable fabrication of sub-10 nm polymer nanopores for DNA analysis

**DOI:** 10.1038/s41378-019-0050-9

**Published:** 2019-04-08

**Authors:** Junseo Choi, Charles C. Lee, Sunggook Park

**Affiliations:** 1 Department of Mechanical & Industrial Engineering and Center for BioModular Multiscale Systems for Precision Medicine, Louisiana State University, Baton Rouge, LA 70803 USA; 2 Department of Comparative Biomedical Sciences, School of Veterinary Medicine, Louisiana State University, Baton Rouge, LA 70803 USA

**Keywords:** Nanofluidics, Nanopores, Nanofabrication and nanopatterning

## Abstract

We present the first fabrication of sub-10 nm nanopores in freestanding polymer membranes via a simple, cost-effective, high-throughput but deterministic fabrication method. Nanopores in the range of 10 nm were initially produced via a single-step nanoimprinting process, which was further reduced to sub-10 nm pores via a post-NIL polymer reflow process. The low shrinkage rate of 2.7 nm/min obtained under the conditions used for the reflow process was the key to achieving sub-10 nm pores with a controllable pore size. The fabricated SU-8 nanopore membranes were successfully employed for transient current measurements during the translocation of DNA molecules through the nanopores.

## Nanotechnology: New fabrication technique shrinks nanopores

Researchers in the United States have developed a method to create nanopores smaller than 10 nanometers in a simple, controllable, cost-effective way. Sunggook Park’s team at Louisiana State University pressed an array of silicon microneedles into a polymer layer to produce pores of around 10 nm. The polymer is then briefly heated to make it reflow, causing the pores to shrink. The size of the nanopores can be controlled by regulating the duration of the reflow process. Using this technique with and without the reflow step, the team fabricated nanopores of 12 nm and 6 nm diameter, respectively, and used them to detect single DNA molecules in a solution passed through the pores. This new approach enables researchers to produce with high throughput nanopore devices with precisely positioned sub-10 nm pores which can be used to detect and analyze individual biomolecules.

## Introduction

Nanopores have been proven to be an important tool to detect and analyze single biomolecules^1^ and their transport phenomena through confined geometries^2^. Charged biomolecules, such as DNA, are electrophoretically driven through a nanopore that is either biological (e.g., α-hemolysin and MspA) or solid-state, and are detected as the transient current is measured during their translocation through the nanopore^3^. While biological nanopores provide well-defined pore sizes, shapes, and chemical composition, solid-state nanopores have emerged in recent years because of their chemical, thermal, and mechanical stabilities and because of the ability to control the pore diameter and location during the fabrication. Most of the sub-10 nm solid-state nanopores used for DNA analysis have been produced in inorganic substrates, such as silicon dioxide^4–7^, silicon nitride^8–12^, and glass capillaries^13–16^, via high-energy-beam nanofabrication tools such as focused ion beam^8,9,11,12^, focused electron microscopy^4–7,10,12,17–19^, and a laser-assisted puller^13–16^. The combination of high-energy-beam nanofabrication tools and inorganic substrates is based on the fact that atomic motion to induce local deformation of inorganic substrates requires irradiation by a high-energy beam. Usually, a two-step process has been employed for their fabrication; initial pores with diameters in the range of tens of nanometers have been produced by the nanofabrication tools mentioned above, followed by shrinking the pore size down to ~ 2 nm in a controllable manner using either high-energy electron/ion beams^6,8,16,20–22^ or atomic-layer deposition^7,23,24^. Recently, a single-step process with a high-energy-beam nanofabrication tool, such as focused ion beam and helium ion milling, was also used to demonstrate sub-10 nm pores in inorganic substrates^25–27^. Despite the successful demonstration of nanopore devices for laboratory-scale DNA analysis, the combination of high-end nanofabrication tools and inorganic substrates limits the scalability of fabricating nanopore devices for high yield production because of the serial nature of the fabrication tools and the rather slow deformation of the inorganic substrates. Recently, nanopores as small as 2 nm in size with sub-nm precision have been achieved via the electric breakdown of an insulating Si_3_N_4_ membrane by an applied voltage^28^. Even though this method does not require a high-end nanofabrication tool, in-plane electrodes still need to be built to apply a voltage, which complicates the device fabrication.

Compared to silicon and glass-based inorganic materials, polymers have low material costs, a wide range of physiochemical properties, surface-modification protocols, and a number of low-cost and high-throughput fabrication methods, as exemplified by nanoimprint lithography (NIL), to shape and reshape structures at the micro- and nanoscale^29^. However, the progress on polymer-based nanofluidic devices for single molecular analysis has been focused on utilizing nanochannel/nanoslit structures rather than nanopores for target applications of DNA stretching, molecular preconcentration, separation by nanochannel electrophoresis/chromatography, and solid-phase reactors, as described in a recent review article by Chantiwas et al.^29^. Nanochannel/nanoslit structures in a polymer substrate have routinely been produced by NIL mostly because the long channel/slit structures do not require high aspect-ratio molding. In contrast, the fabrication of perforated nanopores in a polymer substrate requires extremely high aspect-ratio molding because the thickness of the polymer substrate (or membrane) cannot be reduced by the same ratio as the reduction in the pore size to maintain the mechanical strength of the polymer substrate (or membrane). To our knowledge, sub-10 nm pores in polymer substrates have only been demonstrated via ion track-etching techniques;^30^ a polymer film is irradiated by single energetic heavy ions, followed by chemical etching of the resulting latent tracks. Using ion track-etched nanopores, the ion transport behaviors across nanopore membranes have intensively been studied by the Siwy group for potential applications in nanofluidic electronics, biosensing, separation and single-molecular manipulations^31–34^. The track-etching method in combination with a masking technique enabled the production of a single nanopore in polymers. However, it is difficult to position the nanopore at an exact location of a substrate in a deterministic manner, which is important for scaling up the fabrication of nanopore devices and integrating the nanopore device with additional device components such as nanoelectrodes or electronics. Therefore, high-throughput fabrication of polymer-based nanopore devices using a deterministic fabrication method, such as NIL, is crucial to realize more versatile applications for nanopore devices.

Precise control of polymer deformation at the nanometer scale is the key to the fabrication of sub-10 nm pore devices; however, it is difficult to achieve this at the molding temperature during NIL. Wang et al. reported a process called the pressed self-perfection process, in which polymer nanostructures were pressed by a blank Si wafer at a temperature close to the glass transition temperature^35^. This process not only decreased the width and diameter of the nanoscale trenches and holes, respectively, but also reduced the sidewall roughness of those structures. Recently, we reported an easy, simple, and cost-effective method to generate nanopores in a freestanding polymer membrane using NIL using the pressed self-perfection process^36^. Starting with micropores with 3 μm diameter, the pore size was effectively reduced to ~ 300 nm. However, it was difficult to apply the pressed self-perfection process to fine-control the pore size to be below 100 nm because of the large initial pore diameter and the fast shrinking rate (130–288 nm/min within 5 min depending on the applied pressure). In this article, we report and focus on a new strategy that can achieve sub-10 nm conical nanopores in freestanding polymer membranes to perform a DNA translocation experiment. For the fabrication, NIL was used to produce the initial nanopores, followed by a polymer reflow process to reduce the pore size with fine control.

## Results and discussion

**Fig. 1:**
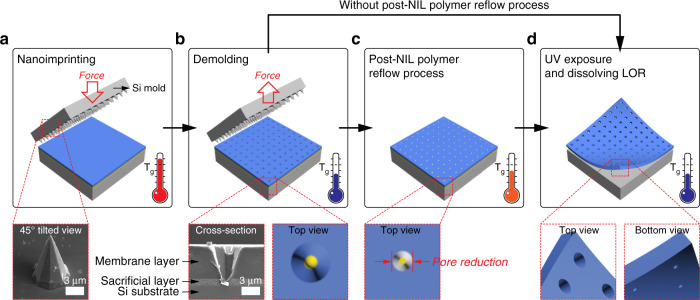
The process of producing perforated nanopores in a freestanding polymer membrane via NIL and polymer reflowing. **a** A silicon microneedle array was molded into a thin double layer of polymer resist. The insert is an SEM image of an Si microneedle. **b** The mold was released from the imprinted substrate. The insert is a cross-sectional SEM image of the imprinted double resist layer. **c** The reflowing process was conducted to further reduce the pore size. **d** The patterned resist was exposed to UV for curing, followed by the release of the membrane from the substrate by dissolving the sacrificial layer. NIL, nanoimprint lithography; SEM, scanning electron microscopy

Figure 1 outlines the process of producing perforated nanopores in freestanding polymer membranes.

The membrane was fabricated by modified NIL with a silicon (Si) microneedle mold (Fig. 1a). The insert in Fig. 1a is a scanning electron microscopy (SEM) image of the Si microneedle mold with a tip diameter in the range of 25 nm, which was fabricated via photolithography and wet chemical etching of Si. The bottom diameter of the microneedles was 5.8 ± 0.2 μm, and the corresponding microneedle height was 9.1 ± 0.1 μm. The Si microneedle mold was imprinted into a thin double resist layer at 65 °C and at 0.1 MPa for 2 min using a commercial nanoimprinter (Obducat, Sweden). The double resist layer consisted of a 100 nm thick sacrificial layer of lift-off resist (LOR) (MicroChem, USA) that was spin-coated on the Si substrate and a 5.5 μm thick SU-8 membrane layer (MicroChem, USA) that was spin-coated over the LOR sacrificial layer. SU-8 was chosen as the active membrane layer because of its high mechanical stability after ultraviolet (UV) curing. The mold was released from the imprinted substrate at a demolding temperature of 40 °C (Fig. 1b). The insert in Fig. 1b is a cross-sectional SEM image of the imprinted double resist layer of 5.5 μm thick SU-8 and 1 μm thick LOR coated on the Si substrate. The imprinting was conducted at 65 °C and 1 MPa for 2 min. One-micrometer-thick LOR was used only for visualizing a cross-sectional view of the SU-8 membrane and LOR layers, and all the conical nanopores shown in this paper were fabricated using a 100 nm thick LOR. The results indicate that not only was the Si microneedle mold faithfully replicated to the membrane SU-8 layer with good replication fidelity but also, under this imprinting condition, no additional window-opening process was required to achieve perforation through the SU-8 membrane layer. A systematic investigation revealed that perforation of the conical nanopore structures through the SU-8 membrane layer directly by NIL depends on the imprint pressure, the thickness of the sacrificial LOR layer, and the mold structures (results not shown). After imprinting, the polymer reflow process was performed for some samples at 45 °C for 1 min (Fig. 1c). This was followed by the exposure to UV light for curing the SU-8 layer and for the release of the nanopore membrane from the substrate by dissolving the sacrificial layer in MF319 solution (Rohm and Hass, USA) (Fig. 1d). The thickness of the freestanding SU-8 membrane was identical to the initial coating thickness of ~ 5.5 μm. Different thicknesses for the SU-8 membrane layer can be selected, in which considerations should include dimensions of mold structures, mechanical stability and handling of the freestanding membrane after lift-off, and the stress generated during spin coating and molding/demolding. The thickness of the SU-8 layer also affects the yield of the molding process because it requires a longer time for mold structures to punch through a thick membrane layer. Based on our experience, the optimal thickness range for the SU-8 membrane layer is 1–10 µm. For the conical nanopore formed in the membrane, we define the larger pore exposed to one side of the membrane as the base pore and the smaller pore exposed to the other side of the membrane as the tip pore (Fig. 1d).

**Fig. 2:**
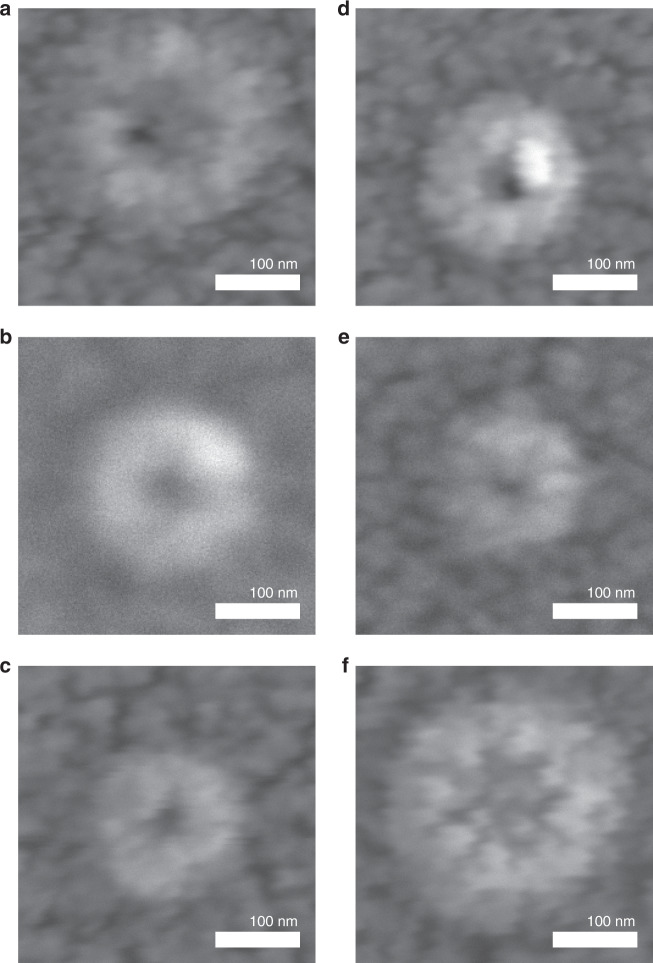
SEM images of the tip pores for nanopore membranes in 5.5-µm-thick SU-8 fabricated via NIL. **a**–**c** Without and **d**–**f** upon undergoing a post-NIL polymer reflow process at 45 °C for 1 min. Occasionally, the pore was blocked after the reflowing process (**f**). NIL, nanoimprint lithography; SEM, scanning electron microscopy

Figure 2 shows the SEM images of the tip pores in the SU-8 membranes for samples that do not (Fig. 2a–c) and that do undergo the polymer reflow process at 45 °C for 1 min after NIL (Fig. 2d–f).

**Fig. 3:**
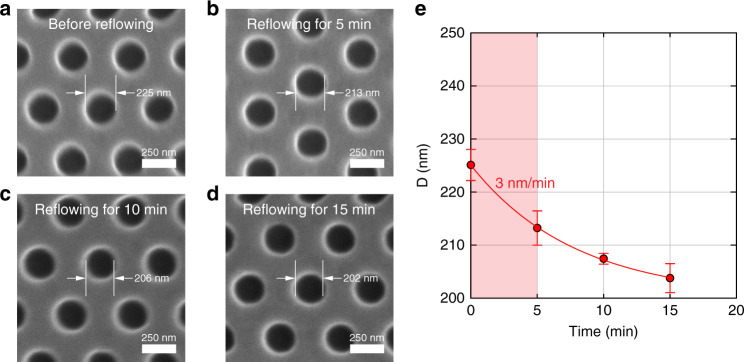
Determination of shrinkage rate for the post-NIL polymer reflow process. **a** An SEM image of an SU-8 membrane with an array of nanopores prior to undergoing the polymer reflow process. **b**–**d** SEM images of the SU-8 membrane shown in (**a**) after undergoing polymer reflow at 45 °C for 5, 10, and 15 min, respectively. **e** The diameter of the nanopores as a function of time for the post-NIL reflow process. The shrinkage rate, i.e., the slope of the curve, decreased with time. The initial shrinkage rate determined from the red zone was ~ 3 nm/min. NIL, nanoimprint lithography; SEM, scanning electron microscopy

While the base pore always had an octagon shape (results not shown), replicated from the shape of the Si mold, the tip pores were slightly oval, which we attributed to the imperfection of the Si wet etching that leads to a slightly asymmetric needle apex, undesired deformation of the SU-8 during demolding, and the relaxation of polymer chains after NIL. The tip pore sizes (e.g., horizontal length × vertical length) were roughly estimated from the inner darkest areas of the SEM images, which were 10 × 10 nm, 11 × 13 nm, and 11 × 10 nm after NIL and before undergoing the polymer reflow process (Fig. 2a–c, respectively). After the polymer reflow process, some nanopores were reduced to 6 × 9 nm and 11 × 9 nm (Fig. 2d, e, respectively). However, one nanopore became blocked upon the polymer reflow process (Fig. 2f). Determination of the pore size by SEM only provided a rough estimation because of the 5 nm thick Au/Pd layer deposited on the SU-8 membrane for SEM analysis. In addition, electron beam irradiation during the SEM measurement may deform the polymer membrane. It was difficult to use transmission electron microscopy to characterize our nanopores because polymers are easily deformed or melted upon irradiation with a high-energy electron beam. Our estimation of the pore size using the SEM images was corroborated by the pore-size determination via conductance measurements, which is described later. The shrinkage rate determined for the reflow process was ~ 2.7 nm/min, which was similar to the shrinkage rate of 3 nm/min, which was independently determined for the submicron pores for the 5 min reflow process at the same temperature (Fig. 3).

The shrinkage rate for the post-NIL polymer reflow process is comparable to the shrinkage rates of 6–16 nm/min^37^ and 1.2–15 nm/min^16^ used for silicon- and glass-based nanopores, respectively, via the irradiation of a high-energy electron beam. Although the polymer reflow process has been used in the fabrication of polymer microelectomechanical systems, such as microlenses^38,39^, our results indicate that this process can also be used to achieve a controllable, low-rate polymer deformation, which is the key to the fabrication of polymer devices with sub-10 nm pores.

The fabrication process that we developed has the potential to produce an array of nanopore devices over a large area; it is worthwhile to discuss the manufacturing tolerance in terms of local variations in pore size (i.e., pore size uniformity) over the membrane area. The local variation in the pore size depends on several factors, including non-uniformity of microneedle structures in Si mold, non-uniformity in the resist thickness by spin coating, and non-uniformity of temperature and pressure during NIL. Among them, the most critical is the non-uniformity in the size of the microneedle structures in the Si mold, which inevitably occurs during wet Si etching because of the inhomogeneous transport of the etching solution and etched materials. The height variation of the microneedle structures with an average height of 9.1 μm over the 2 cm × 2 cm Si substrate amounted to ± 100 nm in this study. Because of the height variation of the microneedles, the pores in the outer area of the membrane were not perforated. Thus, for the membrane with 10 nm pores, we could not obtain manufacturing tolerance. Instead, we measured the manufacturing tolerance for an initial nanopore diameter in the range of 100–300 nm, which was ~ 10% of the initial pore size over a 2 cm× 2 cm area. With a decrease in the initial pore size, the manufacturing tolerance in percentage is expected to increase. Therefore, the production of highly uniform stamp structures is a prerequisite step to achieving highly parallel nanopore devices by using the method in this paper.

**Fig. 4:**
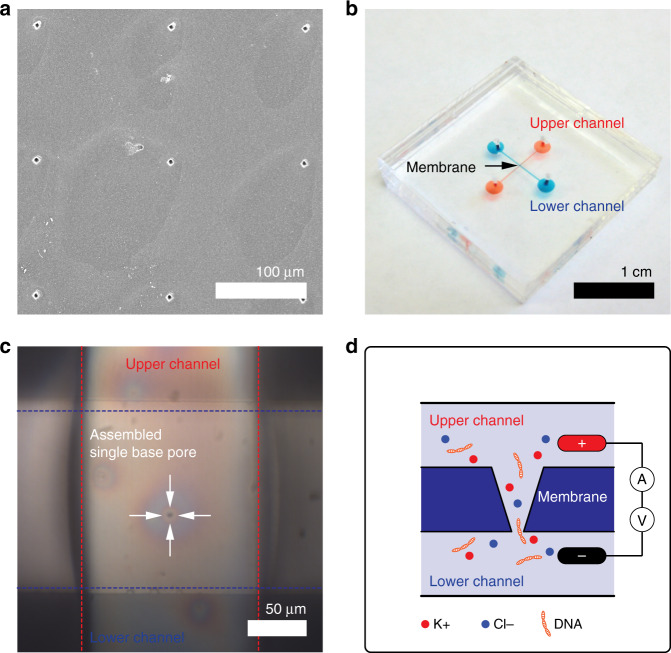
An enclosed nanofluidic device with an SU-8 nanopore membrane. **a** SEM image of base pore patterns with 150 μm pitch between the pores. The average diameter of the pores was 3.6 ± 0.1 μm so that they are visible under a microscope. **b** An enclosed nanopore device consisting of an SU-8 nanopore membrane sandwiched by two PDMS chips with a microchannel in a cross configuration. **c** Optical image of a single base pore located in the crossed area of the two microchannels. **d** A schematic of the setup for DNA translocation experiments. λ-DNA passes through the nanopore from the lower to upper channels (not to scale). SEM, scanning electron microscopy

The fabricated nanopore membrane (without and with undergoing the post-NIL polymer reflow process) was sandwiched between two polydimethylsiloxane (PDMS) chips with a microchannel in a cross configuration to complete an enclosed nanofluidic device for conductance measurements across the nanopore (Fig. 4)^40^.

**Fig. 5:**
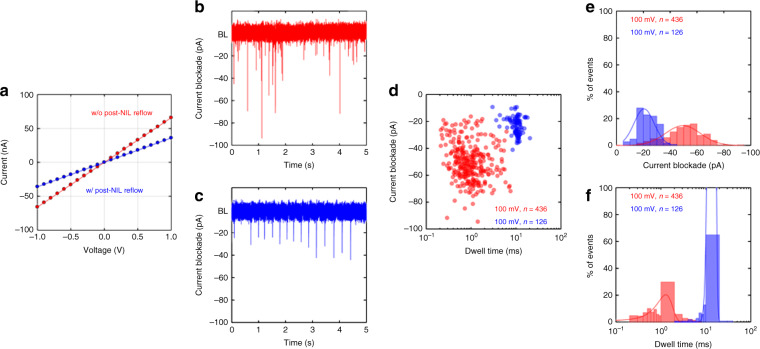
Ion and λ-DNA translocation through an SU-8 nanopore membrane. **a** Current versus applied voltage for two nanopores (a 12 nm pore fabricated via NIL (Fig. 2a) and a 6 nm pore via the combination of NIL and the post-NIL polymer reflow process (Fig. 2d)) in a buffer electrolyte. **b**, **c** Transient current traces showing the translocation of 48.5 kb λ-DNA through two different nanopores at an applied voltage of 100 mV in the same buffer solution. **d** The corresponding event density plot constructed from 436 and 126 translocation events at a 100 mV applied bias. **e** The current blockade histogram and **f** the translocation duration histogram at 100 mV. NIL, nanoimprint lithography

Only one nanopore in the SU-8 layer was located in the crossed area (e.g., 150 × 150 μm) of the two upper and lower microchannels because the pitch of the pore patterns was identical to the width of the microchannels. Supplementary Figure S1 clearly shows that only one nanopore was positioned in the crossed area. Then, the nanopore device was filled with a buffer electrolyte of 1 M KCl (Fluka, USA), 10 mM Tris, and 1 mM ethylenediaminetetraacetic acid (EDTA) (Fluka, USA) at pH = 8.0, and the current–voltage (I–V) curves were measured between two Ag/AgCl electrodes placed across the SU-8 nanopore membrane (Fig. 5a). Like any negative resist, SU-8 is known to swell in the presence of a solvent^41,42^. It has been reported that improved hardness by hard baking makes the SU-8 layer less prone to swelling by the chemicals/buffers that are used for microfluidic applications^41^. In our experiments, after initial disturbance, the conductance values through the nanopores were stable, indicating that the variation in our pore dimensions during the measurements was negligible. One possible explanation is that the SU-8 epoxy swelled and rapidly became saturated upon introduction of the electrolyte solution^42^. For both nanopores, the I–V curves showed a linear behavior without any noticeable ion rectification behavior. The current value for a given voltage (blue circles in Fig. 5a) was significantly lower for the nanopore membrane modified by the reflow process, confirming the decrease in the pore size by the post-NIL reflow process.

Fitting the I–V curve with a linear function in the range of ± 100 mV resulted in conductance values of 66 and 36 nS for the nanopores without and with the post-NIL reflow process, respectively. These conductance values corresponded to 12 and 6 nm in pore diameter, respectively, as calculated from the following formula: *R* = *ρL/πr*_tip_(*r*_tip_ + *L*tan*θ*), where *R* is the resistance of the pore, *ρ* is the resistivity of the solution (obtained from our measured conductivity of 11.06 S/m), *L* is the length of the pore, *r*_tip_ is the tip-pore radius, and *θ* is the conical half-angle estimated from the mold geometries (17.55°)^43^. Kowalczyk and Dekker proposed an improved formula for ionic conductance that included access resistance at the nanopore entrance for an hourglass-shaped solid-state nanopore^44^. The access resistance becomes the dominant contribution at large pore diameters and can be neglected for smaller pore regimes with a pore diameter of less than 15 nm^44^ as in our case. Moreover, our nanopore shape is a conical shape, not an hourglass shape. The formula that we used relates the tip diameter to the corresponding conductance for conical-shaped nanopores and is thus suitable for modeling our nanopore conductance even though the access resistance is not considered in the model. The tip pore diameters obtained from the conductance measurements agree well with those estimated from the SEM images. To our knowledge, this is the first report to demonstrate the fabrication of sub-10 nm nanopore membranes via a low-cost and high-throughput but still deterministic fabrication method, which constitutes a critical step towards scaling up the fabrication of nanopore devices and their versatile applications.

**Fig. 6:**
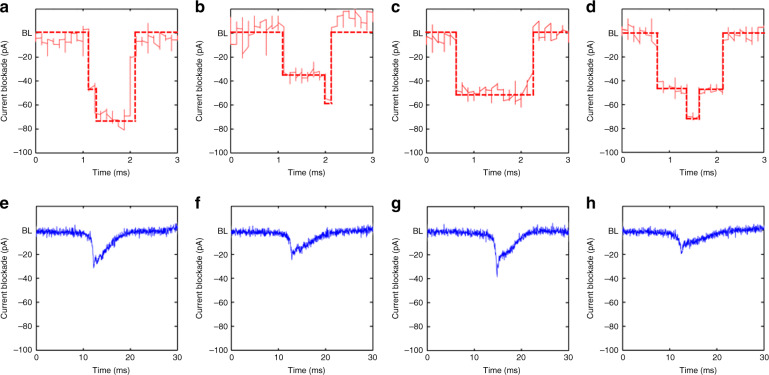
Sample transient current traces zoomed in from Fig. 5b, c. For the 12 nm pore in (**a**–**d**), most peaks look almost symmetric with different translocation modes, such as linear, double local folded, single local folded or fully folded fragments of DNA molecules, whereas peaks for the 6 nm pore in (**e**–**h**) are asymmetric, which we attribute to the larger entropic barrier associated with the smaller pore; the DNA molecule resides at the tip of the pore for a certain duration in a coiled state prior to overcoming the entropic barrier to then thread into the nanopore in a linear fashion

The feasibility of the fabricated polymer nanopores in the SU-8 membrane as single molecular sensors was examined by measuring the transient current during the translocation of DNA molecules through the nanopores. The literature has reported both increases and decreases in ionic pulses upon passage of DNA molecules, depending on the ionic salt concentration of the DNA solution. The current decrease (i.e., current blockade) usually occurs at high ionic salt concentrations because of the occupation of DNA chains within the finite volume of the nanopores/nanochannels^45,46^. The current increase occurring at low salt concentrations is attributed to the enhanced flow of counter ions along the DNA molecular chain^45,46^. For our experiments, a 2 ng/μl λ-DNA (New England BioLabs, USA) solution of the same ionic strength as the previous conductance measurements was added to the reservoir. A positive potential was then applied to the electrode located on the side of the base pore (Fig. 4d). λ-DNAs passed through the nanopore in the direction from tip to base of the pore. Figure 5b, c shows exemplary current traces recorded at 100 mV. All the peaks were downward (i.e., decrease in current), indicating that the decrease in current due to the volume filled by the DNA is dominant over the increase in current due to the mobile counter charge of the DNA molecule^47^. However, the characteristics of the current blockade were different for the two different nanopores (12 and 6 nm). First, the frequency of the peaks (the capture frequency, *f*) decreased with a decrease in pore diameter and with an *f* of 498 ± 62 and 138 ± 12 min^-1^ for the 12 and 6 nm pores, respectively. The larger nanopore captured the DNA molecules 3.6 times more than the smaller nanopore did, as the diameter became doubled. The capture rate dominated by the transport diffusion-limited regime can be expressed as (*πd*^2^*μ*/4*l*)Δ*V* and is accordingly proportional to *d*^2^, where *d* is the diameter of the nanopore, *l* is the length of the nanopore, *μ* is the DNA-free solution electrophoresis mobility, and Δ*V* is the voltage applied to the electrodes^48^. Thus, our results indicate that for both the nanopores used in this study, the DNA capture into nanopores was mainly diffusion limited. Second, the distribution of the magnitude and duration of the current blockade peaks became narrower for the 6 nm pore (Fig. 5d). This indicates that for the larger nanopore, DNA molecules can pass in different translocation modes, such as linear, double local folded, single local folded, or fully folded fragments of DNA molecules, as previously reported in ref. ^49^. DNA molecules seem to translocate through the smaller pores in a more uniform fashion. Third, fitting the peak distribution (Fig. 5d) with a Gaussian function gave their average current decrease (Fig. 5e) and duration (Fig. 5f). The current reduction of 49.4 ± 1.0 pA for the 12 nm pore is 2.4 times larger than the current reduction of 20.5 ± 1.2 pA for the 6 nm pore. The duration of DNA translocation for the 12 nm pore was 1.3 ± 0.1 ms, and it was one-tenth (13.8 ± 0.6 ms) for the 6 nm pore. These results are opposite to those presented in refs. ^16,50^, in which the current drop (or blocked conductance) caused by the DNA molecules increased with smaller pore diameters. Figure 6 shows the magnified current traces for the DNA translocation events.

Most peaks look almost symmetric for the different translocation modes, including linear, double local folded, single local folded or fully folded fragments of DNA molecules, for the 12 nm pore. For the peaks corresponding to the 6 nm pore, however, the sharp initial decrease in the current was followed by a gradual increase in the current. We attribute this behavior to the larger entropic barrier associated with a smaller pore; the DNA molecule resides at the tip pore for a certain duration in a coiled state prior to overcoming the entropic barrier to then thread into the nanopore in a linear fashion. For the 12 nm pore, DNA molecules reaching the tip pore can be instantaneously threaded with little entropic resistance into the pore in various configurations of less-stretched states^51^. Our results confirm that the polymer nanopores prepared via NIL or via the combination of NIL and the post-NIL polymer reflow process are feasible platforms for substituting the existing silicon- and glass-based nanopores for detecting and analyzing single biomolecules. In addition to low-cost and high-throughput fabrication modalities, the polymer nanopores can provide a low material cost, a wide range of physiochemical properties, and surface-modification protocols and low electrical noise^52,53^ compared with silicon- and glass-based inorganic nanopores.

## Conclusions

This work demonstrated the first fabrication of polymer membranes with sub-10 nm pores using a simple, cost-effective, and high-throughput fabrication method. Conical-shaped nanopores in the range of 10 nm diameter could be achieved by single-step NIL with an Si microneedle stamp. To achieve the sub-10 nm pores, however, NIL needed to be combined with the subsequent polymer reflow process to further reduce the pore size. The low shrink rate of several nm/min makes the polymer reflow process extremely attractive for achieving nanoscale controllability for polymer nanomanufacturing. The shrinkage rate can be further controlled by using different reflowing temperatures. We demonstrated pores with a size of as low as 6 nm in the SU-8 membrane, which were successfully used as a platform to sense the translocating DNA molecules through the nanopore via transient current measurements.

## Materials and methods

### Fabrication of silicon microneedles

Silicon wafers (resistivity of 1–10 Ω cm, orientation of (100), thickness of 500 μm) with a 100 nm Si_3_N_4_ layer on each side were used for fabricating the microneedles. The Si microneedles were fabricated using a combination of photolithography and wet-etching techniques. First, an ~ 0.75 μm thick layer of SU-8 (MicroChem, USA) was spin-coated at 1000 rpm for 45 s on the wafer and then baked at 95 °C for 60 s. Photolithography was performed using the designed photomask in a UV exposure station (Quintel, USA) in a class 100 cleanroom. UV exposure was conducted at 80 mJ/cm^2^, and post-exposure baking was followed at 95 °C for 60 s. Then, the wafer was developed with an SU-8 developer (MicroChem, USA) for 60 s, followed by washing with isopropyl alcohol and deionized water. The exposed nitride layer was dry etched to open a window using the reactive-ion etching (Oxford, USA) process. The power, the chamber pressure, and the gas chemistry used were 150 W, 20 mTorr, and 45 sccm SF_6_, respectively. Subsequently, the wafer was transferred to a 35 wt% KOH solution at 80 °C. The KOH solution was prepared by dissolving KOH pellets (Fisher Scientific, USA) in deionized water. After 10 min, the wafer was removed from the etchant, rinsed in water, and dried with N_2_ gas. Prior to imprinting, the silicon surfaces were treated with fluorinated silane (Gelest, USA) in the vapor phase to reduce adhesion to the resin.

### Fabrication of SU-8 membranes

To fabricate the SU-8 membranes, NIL was combined with a sacrificial layer technique. A substrate was spin-coated with a double resist layer. The 100 nm thick LOR layer (MicroChem, USA) was first spin-coated at 3000 rpm for 45 s as a sacrificial layer and then baked at 150 °C for 3 min. On the LOR layer, an approximately 5.5 μm thick SU-8 (MicroChem, USA) layer was spin-coated at 1500 rpm for 45 s, followed by a two-step baking process at 65 °C for 1 min and then at 95 °C for 3 min. NIL was then performed at 65 °C and 0.1 MPa for 2 min with a commercial nanoimprinter (Obducat, Sweden), which allows for both thermal and UV imprinting. The nanoimprinter was equipped with a high-power, flash-type UV lamp (maximum power ~ 1.8 W/cm^2^) with a 250–400 nm wavelength range for UV curing. After imprinting, the polymer reflow process was performed for some samples at 45 °C for 1 min. This was followed by exposure to UV light to cure the SU-8 layer for 10 s. Both the Si substrates coated with LOR/SU-8 and Si molds were not transparent; thus, we conducted UV exposure after NIL. Finally, the membrane with perforated nanopores was released by dissolving the sacrificial layer with MF319 solution (Rohm and Hass, USA).

### Fabrication of micro- and nanofluidic systems

To fabricate simple microfluidic channels (150 μm width, 150 μm depth, and 6 mm length), an SU-8 negative photoresist was patterned on a silicon wafer via photolithography. A layer of SU-8 (MicroChem, USA) resist was spin-coated at 1500 rpm for 60 s, followed by a two-step baking process at 65 °C for 10 min and 95 °C for 10 min. The thickness of the pre-exposed SU-8 was controlled to be 150 μm with a flycutter (Precitech, USA), followed by post flycutting baking at 65 °C for 10 min. Photolithography was performed using the designed photomask in a UV exposure station (Quintel, USA) in a class 100 cleanroom. The exposed wafer was then developed with an SU-8 developer (MicroChem, USA), followed by washing with isopropyl alcohol and deionized water. The SU-8 pattern on the wafer was replicated by casting PDMS (Dow Corning, USA) (10:1 mass ratio of silicone elastomer to curing agent). After curing overnight at room temperature, the PDMS replica was peeled off from the master. One of the PDMS replicas with a microchannel was gently molded onto a substrate that was spin-coated with the PDMS curing agent, which was used as an adhesive material between the two PDMS replicas. With the other PDMS replica, the fluidic inlet and outlet were formed using a hole puncher. The two PDMS replicas were then bonded at 70 °C for 1 h in a vacuum oven after placing the membrane between them. The membrane was treated with an O_2_ plasma (Harrick Plasma, USA) at 18 W for 30 s to improve the wettability of the pores.

### Electrical measurement

The microfluidic system integrated with the SU-8 nanopore membranes was filled with a buffer electrolyte of 1 M KCl (Fluka, USA), 10 mM Tris (Tris(hydroxymethyl)aminomethane), and 1 mM EDTA (ethylenediaminetetraacetic acid) (Fluka, USA) at pH=8.0. The conductivity of the buffer electrolyte was measured by a conductivity meter (Thermo Scientific, USA). The current signal was measured using an Axopatch 700B low-noise current amplifier (Molecular Devices, USA) with Ag/AgCl electrodes. Data were low-pass-filtered at 10 kHz using the built-in 8-pole Bessel filter. The output signal was sent to a Digidata 1322A data acquisition module (Molecular Devices, USA), was digitized at 200 kHz, and recorded using Clampex 10.2 software (Molecular Devices, USA). For DNA translocation experiments, λ-DNA (New England BioLabs, USA) was added to the buffer electrolyte.

## Supplementary information

SUPPLEMENTAL MATERIAL

## Data Availability

The data that support the findings of this study are available from the corresponding author upon reasonable request.
