# Supplementary material for: Scalable fabrication of sub-10 nm polymer nanopores for DNA analysis
Source: Microsyst Nanoeng. 2019 Apr 8;5:12. doi: 10.1038/s41378-019-0050-9 (PMC6453903; doi:10.1038/s41378-019-0050-9)
Supplement: Supplementary file 1 — SUPPLEMENTAL MATERIAL [file 41378_2019_50_MOESM1_ESM.docx]

**Scalable fabrication of sub-10 nm polymer nanopores for DNA analysis**

Junseo Choi^1^, Charles C. Lee^2^, and Sunggook Park^1,*^

^1^Department of Mechanical & Industrial Engineering and Center for BioModular Multiscale Systems for Precision Medicine, Louisiana State University, Baton Rouge, LA70803, USA

^2^Department of Comparative Biomedical Sciences, School of Veterinary Medicine

Louisiana State University, Baton Rouge, LA70803, USA

1^st^ Author: Junseo Choi

Department of Mechanical & Industrial Engineering and Center for BioModular Multiscale Systems for Precision Medicine, Louisiana State University, Baton Rouge, LA70803, USA

E-mail: [jchoi7@lsu.edu](mailto:jchoi7@lsu.edu)

2^nd^ Author: Charles C. Lee

Department of Comparative Biomedical Sciences, School of Veterinary Medicine

Louisiana State University, Baton Rouge, LA70803, USA

E-mail: [cclee@lsu.edu](mailto:cclee@lsu.edu)

* Corresponding Author: Prof. Sunggook Park

Department of Mechanical & Industrial Engineering and Center for BioModular Multiscale Systems for Precision Medicine, 3290M Patrick F. Taylor Hall, Louisiana State University, Baton Rouge, LA70803, USA

Fax: +1 225 578 5924; Tel: +1 225 578 0279; E-mail: [sunggook@lsu.edu](mailto:sunggook@lsu.edu)

**
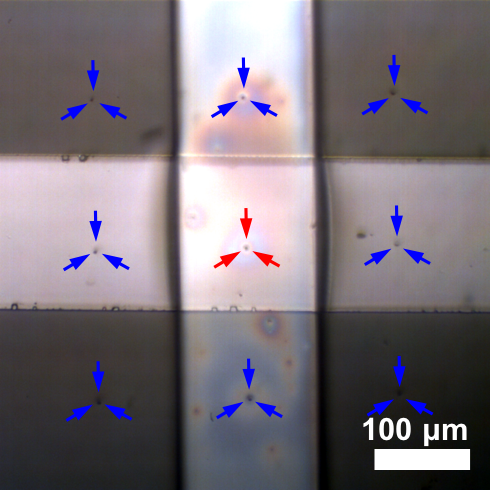
**

**Fig. S1** Optical image of single base pore located in the crossed-area (e.g. 150 μm × 150 μm) of the two microchannels. Red and blue arrows indicate base pores in and out the crossed-areas, respectively. **Target size: Double column fitting image**
